# Experience with the safe admission of breast and thyroid cancer patients in non-endemic areas during an epidemic outbreak

**DOI:** 10.3389/fonc.2023.1220518

**Published:** 2023-09-13

**Authors:** Peng Tang, Xiang Ai, Minghao Wang, Ying Hu, Qinwen Pan, Jun Jiang

**Affiliations:** ^1^ Breast Disease Center, Southwest Hospital, The Army Military Medical University, Chongqing, China; ^2^ Department of Thyroid and Breast Surgery, The General Hospital of Western Theater Command, Chengdu, Sichuan, China

**Keywords:** cancer patient, COVID-19, infectious disease, safe admission, treatment delay

## Abstract

**Background:**

The outbreaks of infectious diseases, such as coronavirus disease 2019 (COVID-19), have seriously affected the normal work and life of the public, as well as the normal diagnosis and treatment of other diseases due to their strong infectivity, high population susceptibility, and diverse clinical manifestations. Breast and thyroid specialists in non-hotspot epidemic areas of COVID-19 must consider factors, including epidemic prevention and control, breast and thyroid cancers and diseases diagnosis and treatment, and access to medical resources to make a reasonable treatment choice and optimize the treatment process.

**Methods:**

A cohort study was designed under our center’s epidemic prevention and control strategy. The study was conducted between February 3 and April 19, 2020, to explore the safety of clinical diagnosis and treatment of breast and thyroid cancer patients during the epidemic. All the outpatients, inpatients, day-time chemotherapy patients, targeted therapy patients, and relevant medical staff in the observation period in the Department of Breast and Thyroid Surgery in Southwest Hospital in Chongqing municipality, China, were included to investigate the detection and infection rate of COVID-19 and suspected patients.

**Results:**

During the observation period, 27,117 patients were admitted to the outpatient unit. We performed 394 inpatient surgeries and 411 day-time surgeries. In our center, 1,046 and 663 patients received day-time chemotherapy and targeted therapy, respectively. All the patients were diagnosed and treated promptly and safely. Three suspected COVID-19 patients were identified in the outpatient unit. Healthcare staff achieved a “zero” infection of COVID-19.

**Conclusion:**

The spread and cross-infection of COVID-19 can be avoided in non-hotspot epidemic areas based on scientific prevention and control, and cancer patients can be diagnosed and treated on time. The prevention and control measure implemented in the COVID-19 epidemic for diagnosing and treating cancer patients was effective and can be referenced for other infectious disease outbreaks.

## 1 Introduction

Coronavirus disease 2019 (COVID-19) has presented a global trend of an explosive epidemic since the first case of new coronavirus pneumonitis appeared in Hubei, China, in December 2019 (1–3). The WHO has declared this COVID-19 outbreak an international public health emergency, and China has classified COVID-19 as a Class B infectious disease under the People’s Republic of China Law on the Prevention and Control of Infectious COVID-19, as well as adopted preventive control measures for Class A infectious diseases (4).

A malignant tumor is a severe disease that threatens human life and health and has become the second-leading cause of death (5, 6). Breast cancer is the first malignant tumor in women with a large patient population (7, 8). Thyroid cancer incidence has rapidly increased recently and has become the first malignant tumor in many countries (9). The primary means of improving malignant tumor survival rates is to strengthen secondary prevention, specifically early detection, diagnosis, and treatment. Several studies have demonstrated that delayed treatment can affect the prognosis of malignant tumor patients (10). Countries and communities have implemented strict traffic control to restrict personnel movements in the context of an outbreak of large-scale infectious diseases represented by COVID-19, and the public is concerned about going to the hospital due to a potential infection. However, COVID-19 patients hiding among ordinary patients may cause nosocomial infections, causing tumor treatment to be delayed. If the outbreak lasts for a long time and the delay in tumor treatment is prolonged, the patient’s survival will be adversely affected. In the context of sudden large-scale infectious diseases, there is an urgent need to develop safety standards for diagnosing and treating malignant tumors, which will guide medical personnel in making scientific diagnoses and treatments of malignant tumors under scientific safeguards.

Based on the Pneumonitis Diagnosis and Treatment Program for New Coronavirus Infection, New Coronavirus Pneumonia Prevention and Control Program, and Coronary Virus Infection Guidelines for Hygiene Protection conducted by the national health committee of the People’s Republic of China, combined with diagnosis and treatment of breast and thyroid malignancies patients, the Breast and Thyroid Disease Center in Southwest Hospital formulated the safe diagnosis and treatment guidelines for breast or thyroid cancer patients in non-high-risk areas during the COVID-19 epidemic. Approved by the ethical review committee, we entered the Chinese Clinical Trial Registry and conducted a prospective observational study in Chongqing to investigate safe methods and preventative measures for treating malignant tumors during an epidemic.

The analysis included all breast and thyroid disease patients admitted to our department during the COVID-19 pandemic. During the nine-week practice, 27,117 outpatient visits and 2,484 inpatients received timely diagnosis and treatment without COVID-19 infection, confirming that safe diagnosis and treatment of breast or thyroid cancer patients during the COVID-19 epidemic can be achieved through enhanced epidemiological screening and ward management. Our practice can provide a reference for diagnosing and treating patients with malignant tumors in the current epidemic environment around the world.

## 2 Patients and methods

### 2.1 Patients

All outpatients and inpatients were included in the Breast and Thyroid Disease Center, Southwest Hospital, and The Army Medical University (Third Military Medical University) between February 3 and April 19, 2020.

### 2.2 Procedures

All patients were screened for COVID-19 in addition to routine diagnosis and treatment measures.

### 2.3 Outpatient screening methods and management

**Figure 1:**
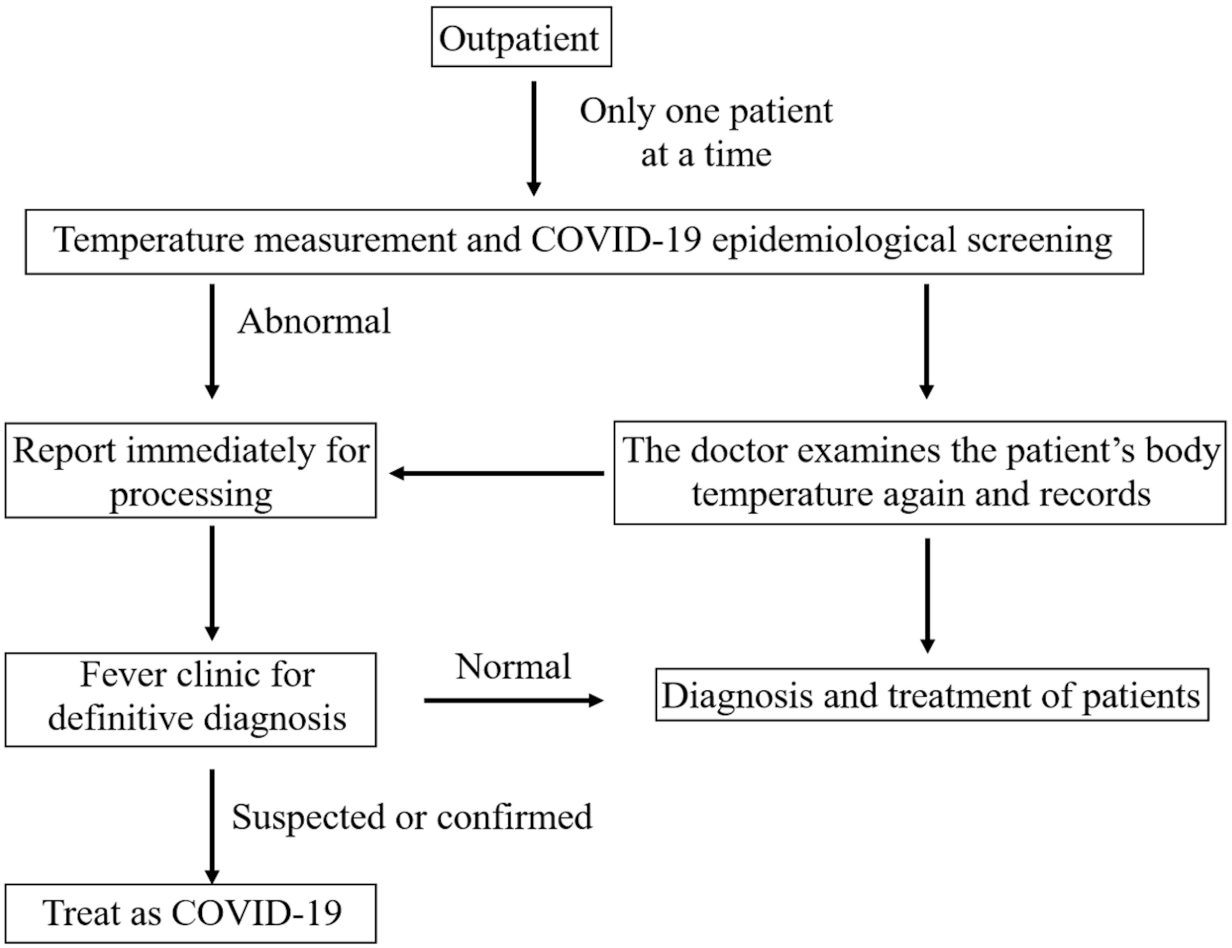
Diagram of Outpatient Screening and Management.

All patients scheduled appointments online, and their body temperature was measured before entering the clinic. Patients with abnormal body temperatures were diverted to the fever outpatient clinic via special channels. Patients with normal temperatures were permitted to enter general outpatient clinics. Before diagnosis and treatment, patients with a “history of traveling or residing in epidemic-prone areas in the last month,” “history of close contact with confirmed or suspected COVID-19 patients in the last month,” “history of close contact with people from COVID-19 hotspot epidemic areas,” and “history of physical discomfort (fever, cough, and other symptoms in the last month)” were excluded. After confirmation of no abnormal body temperature and no high epidemiology risk, the patient was admitted to the specialist clinic in sequence (**Figure 1**).

### 2.4 Inpatient screening methods and management

#### 2.4.1 Admission crowd

Priority treatment was given to patients with breast carcinoma, life-threatening goiter, differentiated thyroid cancer with suspected lymph node metastasis, rapid progression or risk of tumor invasion, and medullary thyroid cancer. Delayed treatment was given to confirmed or suspected COVID-19 patients who were not cured, patients with chest computed tomography (CT) suggested that COVID-19 (admitted after cured or COVID-19 was excluded), patients with close contact history with confirmed or suspected COVID-19 personnel within two weeks (changed to four weeks after 2020-02-10), and patients with a history of living or traveling in COVID-19 high-risk areas and a history of fever or cough (admitted after the observation period).

#### 2.4.2 Pre-hospital screening

Routine examinations were completed before admission to reduce the waiting time before hospitalization. Every patient’s body temperature, blood routine examination, chest CT, and 2019-nCoV nucleic acid examination (2019-nCoV IgM/IgG antibody examination after March 11, 2020) were completed the day before admission. Only one family member who planned to accompany the patient during hospitalization was required to conduct COVID-19-relevant examinations. Patients with no abnormalities in the above examinations were admitted to the hospital.

#### 2.4.3 Patient management and indications

Patients in the outpatient unit were given corresponding clinical examinations based on their primary complaint and physical examination results to diagnose and exclude malignant tumors. Patients with malignant and benign tumors requiring confirmation surgery were admitted to the hospital for surgical treatment. Patients with suspected malignancies requiring biopsy, benign tumors with rapid growth, or patients with great psychological stress were treated with daytime surgery. Breast cancer patients with indications for chemotherapy or targeted therapy were treated with neoadjuvant, adjuvant, or salvage therapy in the daytime ward.

#### 2.4.4 Hospitalization management

**Figure 2:**
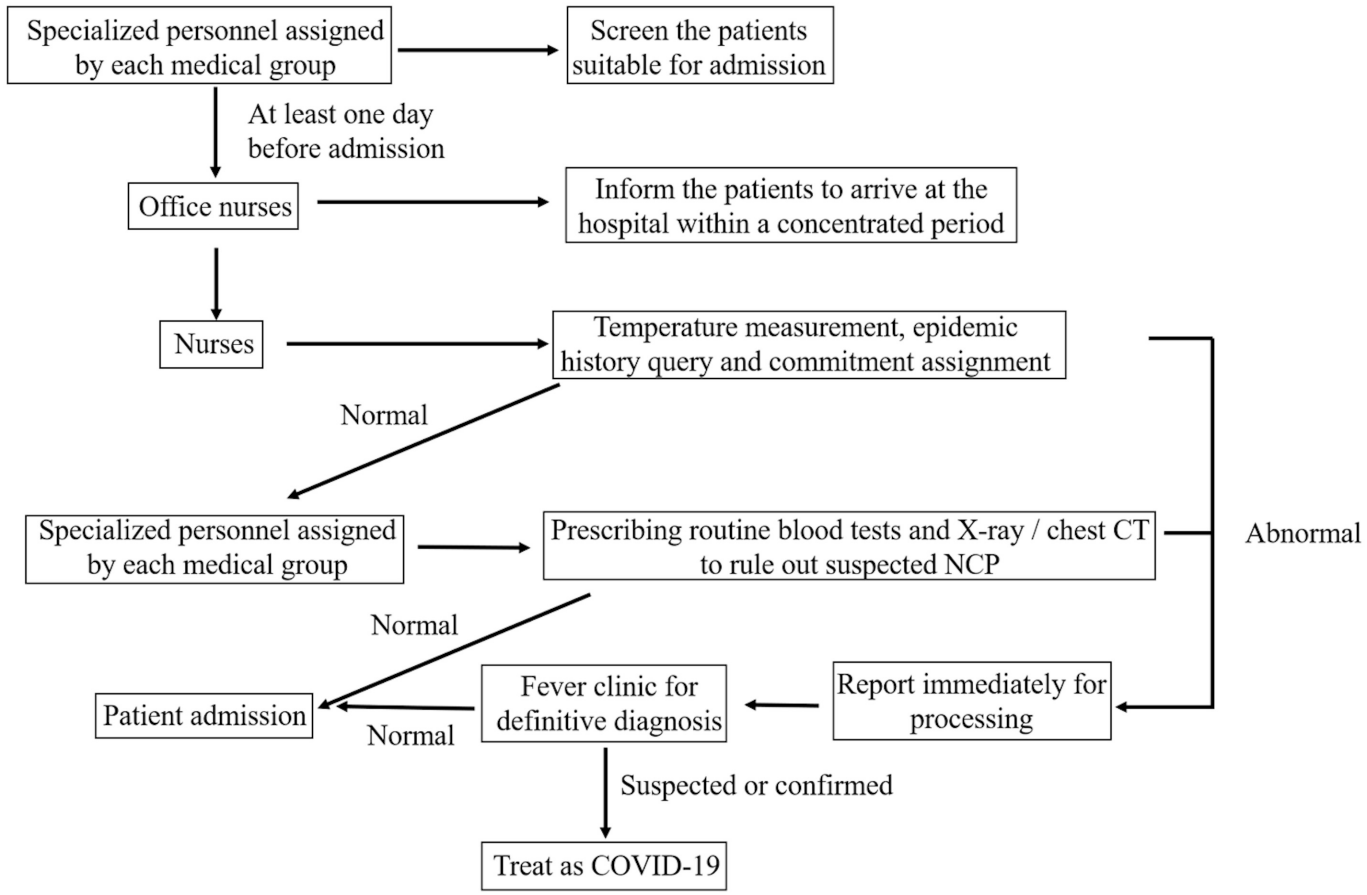
Diagram of Inpatient Screening and Management.

All patients and their families were tested for temperature and COVID-19 epidemiological screening before entering the ward. Patients were admitted to the ward sequentially after confirming no abnormal temperature or high epidemiology risk (**Figure 2**).

All patients were treated in a single room (adjusted to a separate bed after the secondary response to a major public health event on March 11, 2020). Patients and their family members wore masks throughout the process, and their body temperature was measured four times daily. Patients with T > 37.3°C had their surveillance tightened, the cause of their fever was examined, and if required, they were referred to the fever clinic for a precise diagnosis.

The patient’s temperature was measured four to six times daily after surgery. When a patient has a fever, the operation area should be immediately checked for infection, bleeding, and effusion. Febrile patients were asked about their cough, sputum, and breathing. The blood routine, chest CT, and COVID-19 nucleic acid test were conducted to identify the cause of the fever and treat it accordingly.

During the interphase of chemotherapy, the patients should be well prepared for infection prevention and granule deficiency prevention. Anti-infective treatment was conducted if the symptoms of fever appeared. Simultaneously, COVID-19 should be excluded by the fever clinic, and the patients should be hospitalized for treatment according to their conditions.

#### 2.4.5 Protection of medical staff

Before entering the ward, all staff took a temperature reading, monitored it twice daily, and kept a record. Those who were unwell (temperature ≥ 37.3°C, cough, and other symptoms) went to the fever clinic. After entering the ward, all staff members wear first-level protective clothing, disposable isolation gowns, goggles or face shields, and double-layer gloves when performing invasive operations (puncture and extubation). The seven-step hand-washing method was followed by all staff members before and after patient contact. Anesthesiologists wear disposable isolation gowns, goggles or face shields, and double-layer gloves based on first-level protection to isolate the sputum spatters of the patient during endotracheal intubation or extubation.

#### 2.4.6 Ward disinfection

All of the ward’s windows were opened, and the rooms were ventilated two times per day for 30 min each. The following operations were performed: ultraviolet (UV) and negative ion disinfection once a day, 1 h per time; 500 mg/L chlorine-containing spray air disinfection twice a day; and floors, windowsills, and the object’s surface were mopped and wiped with a 1,000 mg/L chlorine-containing preparation twice daily.

The drainage fluid was soaked with 1,000 mg/L chlorine-containing preparations for 30 min and then poured into the hospital’s special sewage system. The contaminated dressings were disinfected with 1000 mg/L chlorine-containing preparations and treated as medical waste. The used quilts were sent to the decontamination room for cleaning and disinfection. Special infections were collected in special bags for recycling and reported to the decontamination room. Bedding and pillows were disinfected with ozone for 1 h and sent to the decontamination room for disinfection and cleaning if contaminated with blood, body fluids, or secretions from patients (11, 12). Ozone is a reliable, clean oxidizing agent with a powerful microbicidal effect against bacteria, viruses, fungi, and protozoa, reacting with the cytoplasmic membrane and breaking lipid components at various bond sites to inactivate microorganisms. Enveloped viruses, such as coronaviruses, might be more sensitive to ozone than non-enveloped viruses due to the interaction of ozone with the lipid layer envelopes (13). Bed sheets, equipment, and facilities were wiped with 1,000 mg/L chlorine-containing preparations. Electrocardiogram (ECG) monitors and oxygen devices were wiped and disinfected with 75% alcohol after use.

#### 2.4.7 Patient follow-up

Patients discharged with a drainage tube were required to remove the tube at the nearest hospital after meeting the tube removal conditions. The blood routine was regularly checked in the nearest hospital for daytime chemotherapy patients. After discharge, the patient was followed up by the office nurse for one month, once a week. The content of the return visit included rehabilitation status and epidemic history.

#### 2.4.8 Outcomes

The primary outcomes are the detection and infection rates of suspected COVID-19 patients among medical staff and patients, whereas the secondary outcome is the therapeutic effect for breast and thyroid disease patients.

## 3 Results

### 3.1 Patient admissions

The Breast and Thyroid Disease Center, Southwest Hospital, The Army Medical University, had 27,117 outpatient visits, 394 inpatient surgical procedures, and 411 day-time surgical procedures from February 3 to April 19, 2020. **Table 1** illustrates that 1,046 and 663 breast cancer patients received day-time chemotherapy and targeted therapy, respectively.

**Table 1:** No. of patient admissions.

Category	Outpatient	Inpatient Surgery	Day-time Surgery	Day-time Chemotherapy	Day-time Targeted Therapy
**Number**	27,117	394	411	1,046	663

### 3.2 Diagnosis and treatment results

All outpatients received necessary clinical examinations and effective treatment. No treatment-related grade 3–4 toxicities occurred in patients who underwent daytime chemotherapy or targeted therapy. None of the 394 patients who underwent inpatient surgery and 411 who underwent day-time surgery had serious operation-related complications.

### 3.3 Patients comparison with last year

**Figure 3:**
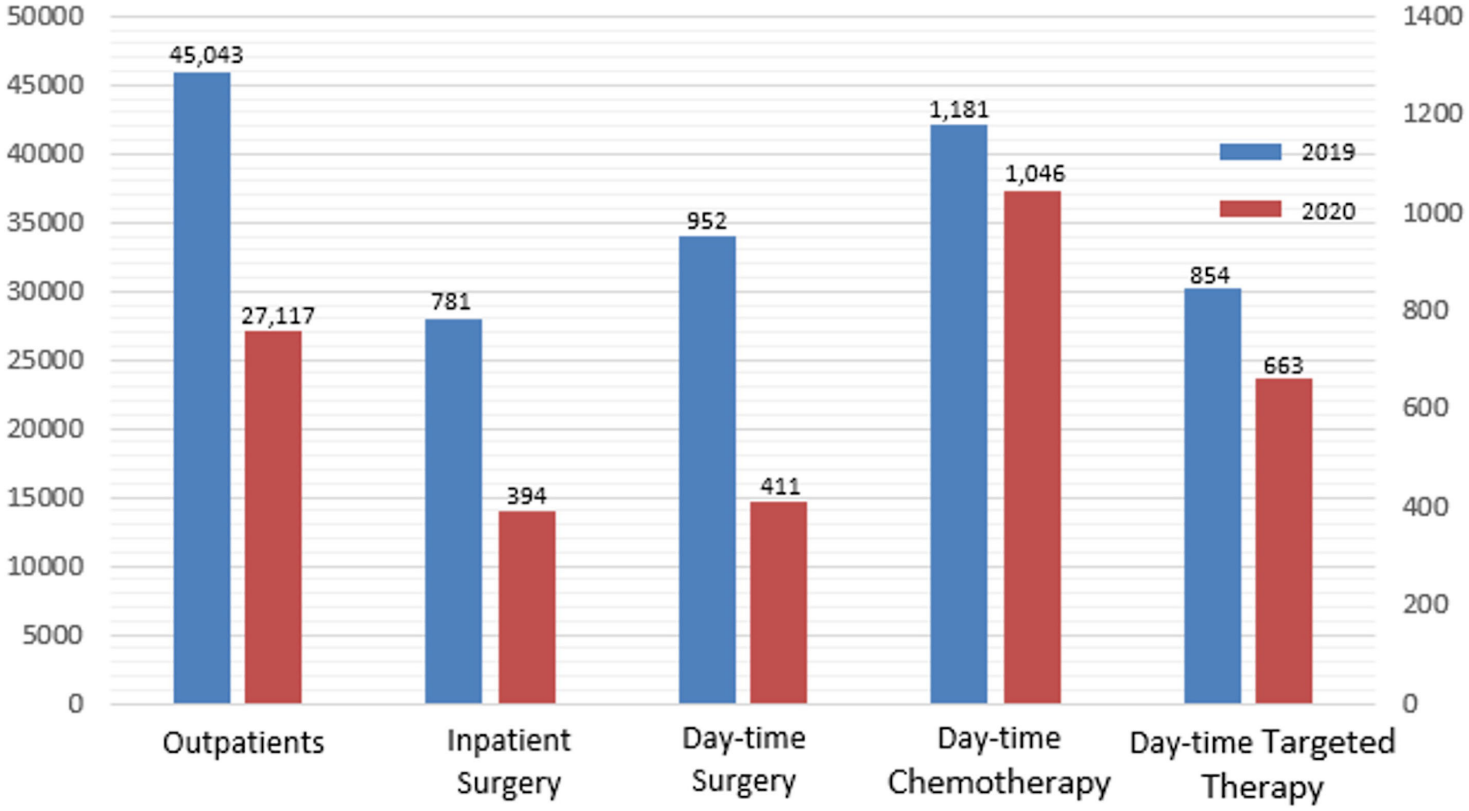
Comparison of the number of patients during the epidemic period in 2020 with the same period in 2019.

**Figure 3** compares the number of patients in the same period last year. The number of outpatient visits, inpatient surgical procedures, day-time surgical procedures, and patients who underwent day-time chemotherapy and targeted therapy decreased significantly compared to last year.

### 3.4 Infection condition of COVID-2019 in medical staff and patients

**Figure 4:**
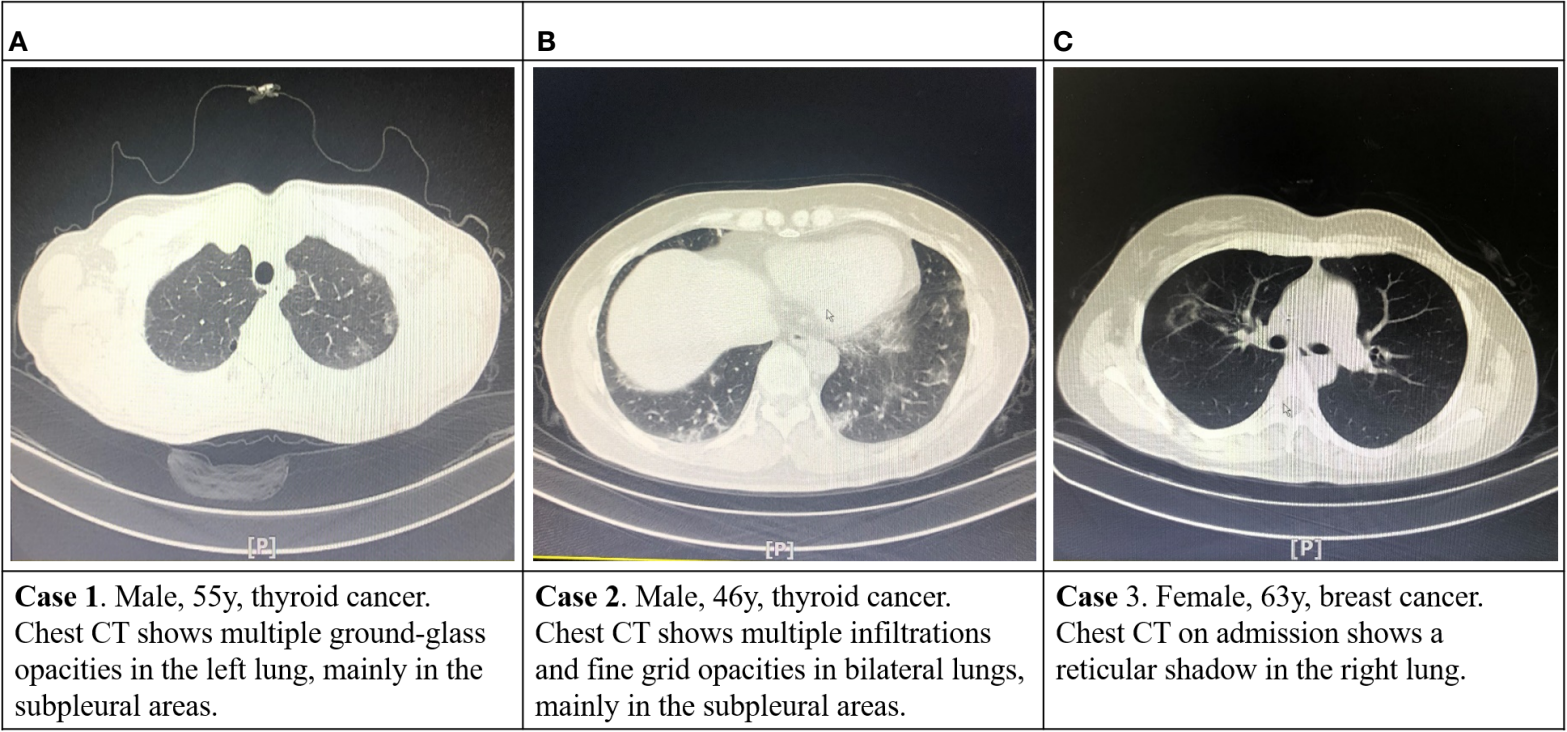
The chest CT images of three suspected asymptomatic NCP infection cases.

Medical staff and patients achieved zero infections with novel coronavirus pneumonia (NCP). Three cases of suspected asymptomatic NCP infection were detected using a chest CT scan in the outpatient unit, and the epidemiology history of COVID-19 was meticulously traced. (**Figures 4A–C**). These patients were immediately instructed to perform a throat swab nucleic acid test (all results were negative) in the fever clinic and were enjoined to be isolated at home and reviewed again after two weeks. All three patients were excluded from the NCP infection two weeks later. The relevant medical staff was present without risk of exposure, and isolation was unnecessary.

### 3.5 Postoperative recovery of surgery patients

After surgery, 36 breast and thyroid cancer patients experienced transient fever (37.1–38.4°C), which was more common in the first one to two days after surgery. The fever usually lasted 4–40 h. The blood routine white blood cells (WBCs) and neutrophile granulocytes (NEUs) were higher than normal and returned to normal without special treatment. Telephone follow-up revealed no obvious signs of fever, and no complications, such as infection or bleeding, existed.

### 3.6 Follow-up results

The WeChat group of patients who underwent surgery or chemotherapy received timely and effective non-contact follow-up on discharged patients. Patients reported a fever condition every day. Patients who received chemotherapy must report the status of their WBCs. The results indicated that all the patients had no abnormal fevers or serious complications. We treated 67 chemotherapy patients with leukopenia in the local hospital or outpatient unit. All patients had no NCP infections at the time of manuscript submission.

## 4 Discussion

According to the epidemic situation, Chongqing adjusted the first-level response to the public health emergency to the second-level response on March 10, 2020. This study’s observation period was conducted in Chongqing, China, during an epidemic.

Previous reports indicate that delayed diagnosis and treatment of patients with malignant tumors may adversely affect the prognosis (14, 15). Some studies have demonstrated the effect of delayed surgery on patient survival (16–19), whereas others have revealed the relationship between delayed adjuvant therapy and the prognosis of cancer patients (20–22). Several studies have indicated that delayed treatment can adversely affect patient survival outcomes. Eriksson et al (18). reported that delayed surgery would affect the overall survival of breast cancer patients. The risk increased by 1.011 (95% CI 1.006–1.017) for each day of delay from diagnosis to surgical treatment. Trufelli et al (23). reported a 1.3-fold (95% CI 1.06-1.71, p = 0.015) increase in overall survival risk for each month of a delay between surgery and postoperative chemotherapy. Timely and effective diagnosis and treatment of malignant tumors, including breast and thyroid cancer and life-threatening breast and thyroid diseases (acute mastitis and giant goiter), are essential during the epidemic.

Epidemic contradictions may conceal other contradictions. For instance, the diagnosis and treatment of many diseases, including malignant tumors, have been delayed (24). During the NCP epidemic period, breast and thyroid cancer patients could not receive timely treatments due to strict traffic controls (25). The public may not go to the hospital because of concerns about possible infection in hospitals, and medical staff may have concerns about potentially infected patients and thus suspend routine outpatient and non-emergency admission. However, the number of patients delayed in hospital presentation and the duration of these delays due to epidemic factors are inestimable. Besides the inability to treat the disease timely, delayed treatment can lead to psychological panic and anxiety in cancer patients, causing new social problems. This is a new problem that must be investigated and solved. There is a lack of evidence in epidemic non-hotspot areas with sufficient medical resources to completely stop the diagnosis and treatment of tumor patients and other patients who must be treated within a time limit. Therefore, several medical professional committees have formulated guidelines for diagnosing and treating diseases during the epidemic to ensure that patients with emergency and serious diseases can receive timely treatment during the epidemic while protecting the safety of medical staff and patients.

There is still a lack of experience in the timely and effective treatment of patients with malignant tumors while preventing the spread of an infectious disease outbreak such as COVID-19. In this study, 27,117 outpatients and 2,484 inpatients received day-time chemotherapy and targeted therapy timely. All breast and thyroid cancer patients who required treatment were admitted timely and infection-free. In this study, 411 patients received day-time surgery. Day-time surgery clarified the diagnosis of suspicious malignant patients and eliminated panic in benign tumor patients. The effect of day-time surgery is safe and reliable. Except for three patients suspected of COVID-19 infection, other patients had no treatment delay or were forced to change their treatment plan. The scientific safety precautions confirm that the adopted infectious disease prevention and control measures are feasible and effective in non-hotspot disease areas.

The tumor can be diagnosed and treated safely under active prevention and control. During the COVID-19 epidemic, tumor patients should be screened and treated at intervals (we treated patients in a single room in the first-level response period), and protection should be strengthened. According to the “New Coronary Virus Pneumonia Diagnosis and Treatment Scheme (Trial Version 7)” issued by the National Health Committee, the diagnostic criteria for suspected cases of COVID-19 are (1) Epidemiological history (i) History of travel or residence in Wuhan and peripheral areas or other case-reporting community within 14 days before sickness; (ii) History of contact with the COVID-19 infected person (positive nucleic acid test) within 14 days before the onset of illness; (iii) History of contact with patients with fever or respiratory symptoms from Wuhan and peripheral areas, or from a community with reported cases; (iv) Cluster cases (two or more cases of fever and/or respiratory symptoms in a small area such as home, office, and school within two weeks); (2) Clinical manifestations (i) fever and/or respiratory symptoms; (ii) the above-mentioned imaging features of the NCP; (iii) the total number of the WBCs and the lymphocyte count are normal or decreased in the early stage of the disease. Patients with any one of the epidemiological histories and any two of the clinical manifestations are diagnosed as suspected cases. Patients without a clear epidemiological history were diagnosed as suspected cases if they met three clinical manifestations. The body temperature and the epidemic history were investigated for each patient. The patients at risk were sent to the fever clinic or enjoined to be isolated at home. After excluding the preliminary risk, given that only 43.8% of COVID-19 patients had a fever on admission, as reported by Zhong’s team (26), we conducted chest CT to screen asymptomatic infected patients, and three suspected patients were finally screened. We performed nucleic acid and antibody tests where possible. The screening procedures followed guidelines to ensure safety.

During the observational study period, there were 27,117 outpatient visits and 19 outpatient doctors. Although we only took temperature measurements and the history of the epidemic area (travel/residential history of the epidemic-prone regions after January 1, 2020) for outpatients, potentially asymptomatic infections may have been missed due to our protection in place, and medical staff safety was guaranteed. Regarding ward management, since patients and their families had been screened for COVID-19 before being admitted to the hospital, it was sufficient to implement first-level protective measures for daily diagnosis and treatment activities. The daily administration of the ward followed the procedures outlined in the study’s methods. Thus, our measures are safe and effective.

According to reports, approximately 47% of COVID-19 patients have a moderate fever of 38–39°C during hospitalization, and 12% have a fever higher than 39°C (26). After monitoring the body temperature of the patients who underwent surgery, this study discovered that most patients would have a short period of low fever ranging from 37.1 to 38.4°C, and the fever usually occurred within one to two days after surgery. There may be an overlap between the two types of fever; therefore, monitoring the body temperature changes in postoperative patients closely is necessary. The cause of the abnormal fever should be identified, and nucleic acid testing should be conducted when necessary to exclude COVID-19 virus infection. Surgical trauma is relatively high in breast or thyroid cancer patients undergoing radical surgery, and most patients will have increased body temperature after the operation, which may be related to traumatic stress.

Although the epidemic has occurred in all provinces in China, COVID-19 did not form a large-scale outbreak except in Wuhan. It was effectively controlled in a short period due to a series of innovative and effective anti-epidemic measures taken by the Chinese government. Hubei province is the central epidemic area of COVID-19, and Chongqing municipality is adjacent to Hubei province. The incidence of COVID-19 in other areas of China, except for Wuhan City, Hubei Province, is similar to that in Chongqing. Additionally, this epidemic situation is similar to the current COVID-19 epidemic in non-hotspot cities in other countries. Our practice and experience with the active treatment of tumor patients in other countries and regions can be referred to under active prevention and control. Our center is one of the largest centers for diagnosing and treating breast and thyroid disease in southwestern China. Outpatients are primarily from the main urban area of Chongqing, surrounding areas of Chongqing, and other areas in China, each accounting for roughly one-third. The number of outpatients and operations in the COVID-19 epidemic period is only one-half of the same period last year. The number of patients increased significantly after the secondary response started, but it still has not reached the level of the same period last year. This may be related to traffic control during the epidemic preventing patients in other regions outside Chongqing from reaching the hospital. From the perspective of social public health, this situation has presented us with new problems that deserve our attention and must be solved, in which both the health of citizens and the development of society need to be considered.

However, the number of outpatients and inpatients decreased significantly compared to last year. Our data present that approximately 40% of outpatients and 50% of inpatients’ treatment may be delayed. This may be related to the strict personnel movement control measures implemented by the Chinese government during the epidemic. These scientific epidemic prevention measures may delay cancer patients’ timely diagnosis and treatment. Doctors, patients, and society should learn from the epidemic and strengthen the management of cancer patients with more effective measures to avoid delaying diagnosis and treatment under special circumstances.

The present study has several limitations. First, there were no subsequent confirmed cases of the NCP among inpatients and outpatients during the research period. Therefore, we could not further verify the validity of the prevention and control measure implemented in our institution. Second, this study did not further analyze the effect of delayed treatment caused by the epidemic, future research is needed in this area.

This study combined the current situation of combating the NCP in non-hotspot areas with diagnosis and treatment guidelines and expert experience on breast cancer and thyroid cancer and has proposed relevant strategies and recommendations for outpatient visits, hospitalization management, and discharge protection for patients with breast and thyroid disease in non-hotspot areas of the epidemic. The practice has proven to be safe and effective. The method can be adopted as a reference by breast and thyroid specialists throughout the country and the world when the next global infectious epidemic breaks out.

## 5 Conclusions

In conclusion, patients with malignant tumors in non-hot spot epidemic areas can be treated in time under safe protection. The protection experience of our center can be used for communication and reference by medical peers all over the world. On the other hand, since the outbreak of a new infectious disease, such as COVID-19, is brand new to the whole world, many clinical and social public health problems brought about by it are also unprecedented and require further research.

## Data availability statement

The original contributions presented in the study are included in the article/Supplementary Material. Further inquiries can be directed to the corresponding author.

## Ethics statement

This study was approved by the Ethics Committee of the First Affiliated Hospital of Army Medical University, PLA with the clinical registration number ChiCTR2000029909.

## Author contributions

PT and XA contributed equally to writing and revising the manuscript. MW and YH interpreted the data and assisted in writing. QP collected the data. JJ designed the study and drafted the manuscript. All authors contributed to the article and approved the submitted version.
